# Stress of emergency physicians during helicopter operations: impact of patients’ diagnoses, severity of diagnoses, and physicians’ work experience

**DOI:** 10.1186/s12873-023-00786-x

**Published:** 2023-02-20

**Authors:** Katja Petrowski, Camila Paola Malkewitz, Christian Schöniger, Mark Frank, Lorenz Theiler

**Affiliations:** 1 Medical Psychology & Medical Sociology, University Medical Center of the Johannes Gutenberg - University Mainz, Duesbergweg 6, 55128 Mainz, Germany; 2 Central Emergency Admission, Städtisches Klinikum Dresden, Dresden, Germany; 3 Department of Anesthesiology, Kantonsspital Aarau, Aarau, Switzerland; 4 Department of Internal Medicine III, Dresden University of Technology, Dresden, Germany; 5 German Air Rescue, DRF Stiftung Luftrettung gAG, Filderstadt, Germany; 6 Swiss Air Rescue, Rega, Zurich, Switzerland

**Keywords:** Stress load, Heart Rate Variability (HRV), Emergency Physicians (EPs), Helicopter Emergency Medical Service (HEMS)

## Abstract

**Purpose:**

Emergency physicians are in danger of developing illnesses due to stress in their demanding work environment. Until today, scholars have not identified stressors or resilience factors that qualify to promote the preservation of emergency physicians’ well-being. Therefore, potential influencing variables such as patients’ diagnoses, the severity of diagnoses, as well as physicians’ work experience have to be considered. The present study aims at investigating emergency physicians in the Helicopter Emergency Medical Service (HEMS)’ autonomic nervous system activity during emergency operations in one shift with respect to patients’ diagnoses, severity of diagnoses, and physicians’ work experience.

**Methods:**

Measurement of HRV (employing the parameters RMSSD and LF/HF) for 59 EPs (age: *M* = 39.69, *SD* = 6.19) was performed during two complete air-rescue-days, the alarm and landing phase being investigated in particular. Besides patients’ diagnoses, the National Advisory Committee for Aeronautics Score (NACA) was included as an indicator for severity. Diagnoses’ and NACA’s effect on HRV were examined using a linear mixed model.

**Results:**

Both HRV parameters indicate a significant decrease of the parasympathetic nervous system as a function of the diagnoses. Furthermore, high NACA scores (≥ V) predicted a significantly lower HRV. In addition, a lower HRV/RMSSD with increasing work experience was observed as well as a positive association between physicians’ work experience and sympathetic activation (LF/HF).

**Conclusion:**

The present study showed that pediatric diagnoses as well as time-critical diagnoses are most stressful and have the highest impact on the physicians’ ANS. This knowledge allows the development of specific training to reduce stress.

**Supplementary Information:**

The online version contains supplementary material available at 10.1186/s12873-023-00786-x.

## Introduction

Over the past years, numerous international studies have shown that the stress load and stress-related illness rate of emergency service employees are exceptionally high [1]. However, there has been little research about the acute stress load of emergency physicians (EPs) in the Helicopter Emergency Medical Service (HEMS) and its effect on health. Here, the number of operations in one shift has been examined in a previous study as a potential stressor [1]. It did not lead to an increase in stress or changes in the activity of the autonomic nervous system (ANS) [1]. Other stressors and resilience factors have not been explored yet. Thus, for a deeper understanding of the stress load, other potential stressors have to be considered. To this end, the objective stress load caused by patients’ different diagnoses on each operation and the severity of these diagnoses need to be further investigated. Moreover, the physicians’ work experience may function as a resilience factor in their demanding work environment. A deeper understanding of specific stressors and resilience factors for emergency physicians in the HEMS is particularly important to derivate preventive measures, develop training programs, and structure their work accordingly to sustain the EPs’ health. Moreover, identifying specific stressors and resilience factors for EPs in the HEMS may benefit the identification of stressors in other domains of emergency medicine and healthcare in general.

So far, there have only been three articles focusing on the response of the autonomic nervous system of the EPs in the HEMS [1] [2] [3]. Thus, little is known about EPs’ objective stress response. In general, scholars classify emergency operations in the six phases: alarm, landing on the operation side, flight to the hospital, hospital, return flight, and operation end [2]. The baseline is often defined as the seventh phase and operationalized as 3 min before the alarm [1]. Exploring the heart rate (HR) during the different phases of emergency operations in the rescue helicopter, the highest HR values were reached during the alarm phase [2] [3]. An elevated heart rate has been shown to be associated with psychological stress [4] as well as physical exercise [5]. This indicates high strain, especially in the alarm and the landing phase. Besides the HR, heart rate variability (HRV) serves as a parameter for the activity of the autonomic nervous system [6]. A high HRV is a clinical indicator of fast adaptability to external and internal stressors and is a sign of a healthy organism. Conversely, chronic stress can lead to a reduced HRV [7].

Scholars have shown that EPs in the HEMS have lower Heart Rate Variability (HRV) values compared to standard values [1]. Furthermore, the illness rates of employees of the emergency service are exceptionally high [8]. Yet, we do not know what variables specifically function as stressors or resilience factors. There is only one study that examined the number of operations per shift as a potential stressor and could not find this hypothesis confirmed [1]. To derivate adequate preventive measures to sustain the EPs’ health, it is crucial to identify more specific stressors or resilience factors. 

Therefore, the aim of the study is to identify such stressors and resilience factors. Specifically, our objective is to investigate EPs’ physiological stress levels depending on patients’ diagnoses during emergency operations as well as the severity of these diagnoses as potential stressors. Moreover, the EPs’ work experience may serve as a resilience factor. Based on the literature [1] [2] [3], the focus was on the alarm and landing phase due to EPs’ higher sympathetic activity. We postulate that physiological stress in the first phases of the operations increases more strongly with pediatric diagnoses (H1) or time-critical diagnoses (H2) as compared to patients with routine issues (i.e., transfer). Here, the number of years in the job might be a resilience factor for the stress level in the different missions. Specifically, we hypothesize that the more years of experience in the job, the less physiological stress is activated (H3).

## Materials and methods

### Study design and procedure

This study was a field study with a within-subjects design and the objective to measure EPs’ physiological stress load on air rescue days. Across all EPs, two workdays consisting of up to eight emergency operations per team of EPs were examined, with 18 emergency operations in total being analyzed for this study. The dates and status messages from all examined emergency operations were sorted into discreet, 3 min phases using data provided by DRF Luftrettung and Swiss Air-Rescue Rega. Baseline HRV values for Phase 1 were operationalized as the time three minutes before the alarm until the alarm was answered, regardless of the EPs’ immediate activity. The second phase was defined as the first 3 min after the alarm, which is used to put on the last protective equipment and get to the operation field. The third phase was defined by the time in the air including medical as well as landing preparations. Furthermore, to assess subjective acute and chronic psychological distress, the Trier Inventory of Chronic Stress (TICS [9]) and the Symptom Checklist-90-R (SCL-90-R [10]) were filled out by participants and collected shortly before the day of testing.

The implementation and analysis of the HRV was performed pursuant to recommendations by the Task Force of The European Society of Cardiology (ESC) and The North American Society of Pacing and Electrophysiology (NASPE) [11]. The stress load of every emergency operation or activity was rated on a visual analog scale (VAS) from 1 (low) to 6 (high).

The investigation was carried out in accordance with the Declaration of Helsinki and all subjects gave informed consent. The study protocol was approved by the local Ethics Committee of the Medical Faculty of the Technical University of Dresden, Germany (No#EK348092011).

### Heart Rate Variability (HRV)

The HRV was monitored as an objective parameter of the activity of the ANS with its sympathetic and parasympathetic proportions during emergency operations. We employed the RMSSD as a measure for the time domain of HRV and LF/HF as a measure for the frequency domain of HRV. RMSSD indexes the parasympathetic activity as they reflect the variations in vagal sinoatrial control in the respiratory frequency range [6]. High values in RMSSD are associated with a high HRV, while low values show a depression of the HRV as would be the case in stress or chronic diseases [6]. Opposing to RMSSD, high values in LF/HF as a parameter of the frequency domain are associated with a suppressed HRV as would be the case in states of stress [6]. LF displays the influence of the sympathetic nervous system while HF is associated with the parasympathetic nervous system [6]. High values in LF/HF indicate a sympathetic overweight, while low values indicate a growing influence on the parasympathetic nervous system [11]. The HRV was recorded with the monitoring system BioHarness™ 3 [12] during air rescue days. For this, the EPs wore a chest strap with an integrated sensor. The sampling rate of the device amounts to 250 Hz. Subsequent to the HRV measurement the BioHarness™ Log Downloader software was used to conduct the readout process of the stored raw data from the measuring devices. For the calculation of the HRV parameters, the Polar ProTrainer 5™ software [13] was used with automatic filter settings at moderate (minimum protection zone: 6 sqm) to eliminate artifacts from the measured data. For the spectral analysis, autoregressive modeling was used. The duration of the segments was set to three minutes to avoid overlaps and to ensure comparability according to the standards of the ESC and NASPE [11].

### Operation diagnoses

Analysis of the influence of operation diagnoses on psychological stress load was performed based on two indices. Firstly, six clusters of diagnoses were developed, *stroke* (including inter alia transient ischemic attack or intracranial bleeding), *cardiovascular diseases* (CVD; angina pectoris, arrythmia, pulmonary embolism, other CVDs), *child accidents*, *trauma* (e.g., polytrauma or traumatic brain injury), *respiratory incidents* (such as dyspnea) and *other diagnoses* (i.e., rare ones). To compare these clusters, transfer served as a reference due to its predictability. Thus, stress levels may be modest at best.

Moreover, the severity of each operations incident was assessed. For this purpose, we employed the NACA scale for prehospital severity ratings [14]. The scale allows for descriptive classification of patients’ injuries as one of seven groups of severity. It has been shown to have good predictive and construct validity, being strongly correlated with mortality and transfer to intensive care units (ICUs) as well as moderately correlated with the length of ICU and hospital stays [15]. In this study, none-life-threating NACA groups (0- III) were aggregated into one group and severed as a reference.

### Psychological assessments

To provide a comprehensive participant description, we assessed some psychological variables. Two test instruments were used to measure the perceived chronic stress and anticipatory cognitive appraisal of the EPs’ workload:

(1) The TICS, a psychological instrument constructed by Schulz and Schlotz [9] was originally derived from the work of Richter and Hacker [16]. It consists of 57 items with a five-point rating scale. It provides nine interrelated factors for the retrospective evaluation of psychosocial chronic stress over the last three months [17]. The TICS has a good reliability and proven validity [17].

(2) Additionally, we used the SCL-90-R [10], a general questionnaire screening for psychiatric symptomatology. It contains 90 items, each on a five-point rating scale. Here, zero represents the patient not experiencing a given symptom at all. In contrast, four represents the patient experiencing a given symptom very strongly. For the SCL-90-R, good reliability and validity has been reported with Cronbach’s α between α = 0.75 and α = 0.88 [18].

### Statistical analysis

To measure the effects of the various explanatory variables during air rescue days on the activity of the ANS, the HR and HRV RMSSD, low-to-high frequency ratio (LF/HF), and SDNN were recorded and served as dependent variables. Mean values (*M*) and standard deviation (*SD*) were calculated for all dependent variables. RMSSD and LF/HF were transformed using logarithm naturalis. Prior to further inferential statistical analyses, we demonstrated that the data was approximately normally distributed.

To assess the effect of operation diagnoses, the severity score (NACA) and work experience in years on RMSSD and LF/HF we employed a linear mixed model (LMM) as recommended when analyzing nested data [19], [20]. Here, RMSSD and LF/HF repeated measurement points were nested in participants as level 2 variables. The time variable had three strata: day, operation, and phase (i.e., three minutes before alarm onset as baseline, alarm, and landing phase). We included a random effect for participants (random-intercept term) to allow the intercept to vary across participants. The effect of operation diagnoses, NACA, and work experience (fixed-effect level 1 variables) on RMSSD and LF/HF (response variables) was then analyzed. We included all factors into one model. All repeated measurement variables were included in each analysis. To avoid redundancy, only results of the first analysis were displayed. Coefficients of the subsequent analyses were similar, but not identical. The LMM computation was performed using 16.1 Jamovi [21].

## Results

### Study participants

All participants were crewmembers of the HEMS DRF Luftrettung at the Dresden Christoph 38 site and Swiss Air-Rescue Rega. On average, *M =* 3.76 (*SD* = 1.56) emergency operations were conducted daily by the DRF Luftnotrettung at their site in Dresden [22] and *M =* 1.97 (*SD* = 0.78) by the Swiss Air-Rescue Rega. Overall, *N* = 40 male and *N* = 19 female EPs took part and completed the study. Participants’ mean age was *M =* 39.69 (*SD =* 6.19) and their mean work experience encompassed 11.37 years (*SD* = 4.66). Moreover, *n* = 43 of EPs were anesthetists, *n* = 6 were surgeons, and *n* = 10 had other specializations. For further information, see Table 1. Participation in the study was voluntary.

**Table 1:** Description of participant characteristics

	Emergency physicians	Norm Sample
Total, n	59	
Females, n (%)	19 (32.2%)	
Age (years), M (SD)	39.69 (6.19)	
BMI (kg/m²), M (SD)	26.52 (3.23)	
Smoker, n (%)	6 (10.17)	
Cigarettes/day, M (SD)	7.67 (3.51)	
Contraceptive pill, n (%)	0 (0)	
Medication intake, n (%)	3 (5.08)	
Work experience (years), M (SD)	11.37 (7.91)	
Medical speciality: Anesthesiology Surgery Other	43 6 10	
*SCL-90-R*		
GSI, M (SD)	0.17 (0.18)	0.36 (0.37)
PST, M (SD)	12.04 (12.42)	22.20 (18.09)
*TICS scales*		
Work overload, M (SD)	14.49 (6.57)	9.77 (6.22)
Social overload, M (SD)	13.20 (3.65)	7.42 (4.98)
Pressure to perform, M (SD)	19.34 (5.57)	12.24 (7.08)
Work discontent, M (SD)	7.92 (3.84)	9.67 (5.57)
Excessive demands at work, M (SD)	5.00 (3.40)	5.57 (4.26)
Lack of social recognition, M (SD)	4.39 (3.06)	4.51 (3.21)
Social tensions, M (SD)	4.68 (3.47)	6.08 (4.53)
Social isolation, M (SD)	4.78 (3.67)	7.11 (5.01)
Chronic worrying, M (SD)	3.83 (2.24)	4.79 (3.35)
Chronic Stress Screening Scale, M (SD)	12.90 (6.99)	13.33 (8.32)

*Note.* TICS norm sample from Petrowski et al. (2012); SCL-90-R Norm Sample from Franke (1995). BMI = Body Mass Index; GSI = Global Severity Index; PST = Positive Symptom Total; SCL-90-R = Symptom Checklist-90-R; TICS = Trier Inventory of Chronic Stress

All participants reported being in good health. HRV-influencing factors such as smoking, medication, oral contraceptives, etc. were assessed as part of participants’ self-reported medical history.

### Effects on RMSSD

The random-intercept, fixed-slope models including the repeated measurement variables day, operation, phase as well as diagnoses, NACA, and work experience on RMSSD showed improved model fit indices compared to the null model. Compared to the null model (AIC = 920.76), the Akaike information criterion indicated an improved model fit (AIC_Full Model_ = 804.63). In all models, the fixed effect Omnibus test (see Table 2) revealed a significant main effect of day, operation, and phase. The fixed effect parameter estimates (see Table 3) showed that HRV increased on day two. Moreover, they indicated that operations at the beginning of the day seemed to be more stressful. Regarding phase, the fixed effect parameter estimates suggested significant differences between the baseline phase and the landing phase – the latter appearing to have been more stressful.

**Table 2:** Results of the Fixed Effect Omnibus Tests of Day, Operation, Phase, Operation Diagnoses, NACA, and Work Experience in Years on RMSSD

	F	Num df	Den df	*p*
Day	5.70	1	448.63	0.017*
Operation	2.19	8	445.52	0.027*
Phase	16.70	2	420.16	< 0.001***
Operation Diagnoses	4.89	6	463.14	< 0.001***
NACA	9.78	4	457.91	< 0.001***
Work Experience in Years	8.15	1	55.92	0.006**

*Note*. p ≤ .05*. p ≤ .01**. p ≤ .001***; NACA = National Advisory Committee for Aeronautics Score; RMSSD = Root Mean Square of the Successive Differences

**Table 3:** Results of the Fixed Effect Parameter Estimates of Day, Operation, Phase, Operation Diagnoses, NACA, and Work Experience in Years on RMSSD

Names	Estimate	SE	df	t	*p*
(Intercept)	2.79	0.11	167.24	25.44	< 0.001***
Reference	Day 1				
Day 2	0.18	0.08	448.63	2.39	0.017*
Reference	Operation 1				
Operation 2	-0.20	0.07	463.76	-2.85	0.005**
Operation 3	-0.19	0.08	455.98	-2.37	0.018*
Operation 4	-0.12	0.08	454.36	-1.46	0.146
Operation 5	-0.34	0.12	447.07	-2.87	0.004**
Operation 6	0.06	0.13	443.95	-0.45	0.656
Operation 7	-0.03	0.15	446.32	-0.18	0.857
Operation 8	0.22	0.24	450.39	0.89	0.373
Operation 9	-0.03	0.33	444.41	-0.08	0.935
Reference	Phase 1				
Phase 2	-0.12	0.05	420.16	-2.24	0.026*
Phase 3	-0.30	0.05	420.16	-5.73	< 0.001***
**Operation Diagnoses**:					
Reference	Transfer				
Stroke	-0.44	0.13	463.02	-3.33	< 0.001***
CVD	-0.26	0.11	471.22	-2.42	0.016*
Child	-0.54	0.12	457.41	-4.33	< 0.001***
Trauma	-0.40	0.10	458.29	-4.12	< 0.001***
Respiratory	-0.46	0.19	463.84	-2.38	0.018*
Other	-0.40	0.09	449.27	-4.30	< 0.001***
**NACA ** **Life Threatening**:					
Reference	No				
Possibly	-0.14	0.07	466.90	-2.08	0.038*
Definitively	-0.18	0.07	465.26	-2.61	0.009**
Reanimation	-0.70	0.13	459.43	-5.41	< 0.001***
Death	-0.97	0.25	452.60	-3.92	< 0.001**
**Work Experience** **in Years**	-0.03	0.01	55.92	-2.86	0.006**

*Note*. p ≤ .05*. p ≤ .01**. p ≤ .001***; NACA = National Advisory Committee for Aeronautics Score; RMSSD = Root Mean Square of the Successive Differences

A significant main effect of the operation diagnoses was indicated by the fixed effect Omnibus test (see Table 2). Specifically, the fixed effect parameter estimates (Table 3) revealed that all operation diagnoses significantly differed from a transfer (Fig. 1a). In particular, operation diagnoses concerning children appeared to have the smallest *p*-value as well as the lowest coefficient (*β*_Child–Transfer_ = − 0.54, *SE* = 0.12,

*t*_(457.41)_ = -2.42, *p* < .001), followed by strokes (*β*_Stroke–Transfer_ = − 0.44, *SE* = 0.13, *t*_(463.02)_ = -3.33,

*p* = < 0.001) and trauma (*β*_CVD–Trauma_ = − 0.40, *SE* = 0.10, *t*_(458.12)_ = -4.12, *p* < .001).

The main effect of the severity of the diagnoses (NACA) reached significance in the fixed effect omnibus test as well (Table 2). Life-threatening diagnoses, reanimations and death significantly showed to predict a lower RMSSD (see Fig. 1b; Table 3). The model explained 17% of the variance within the fixed effects and 64% of the variance including the participants as random effect.

Furthermore, work experience in years showed a significant fixed effect Omnibus test (Table 2). The fixed effect parameter estimates suggested a lower HRV with increasing work experience (Fig. 1c).

**Fig. 1a:**
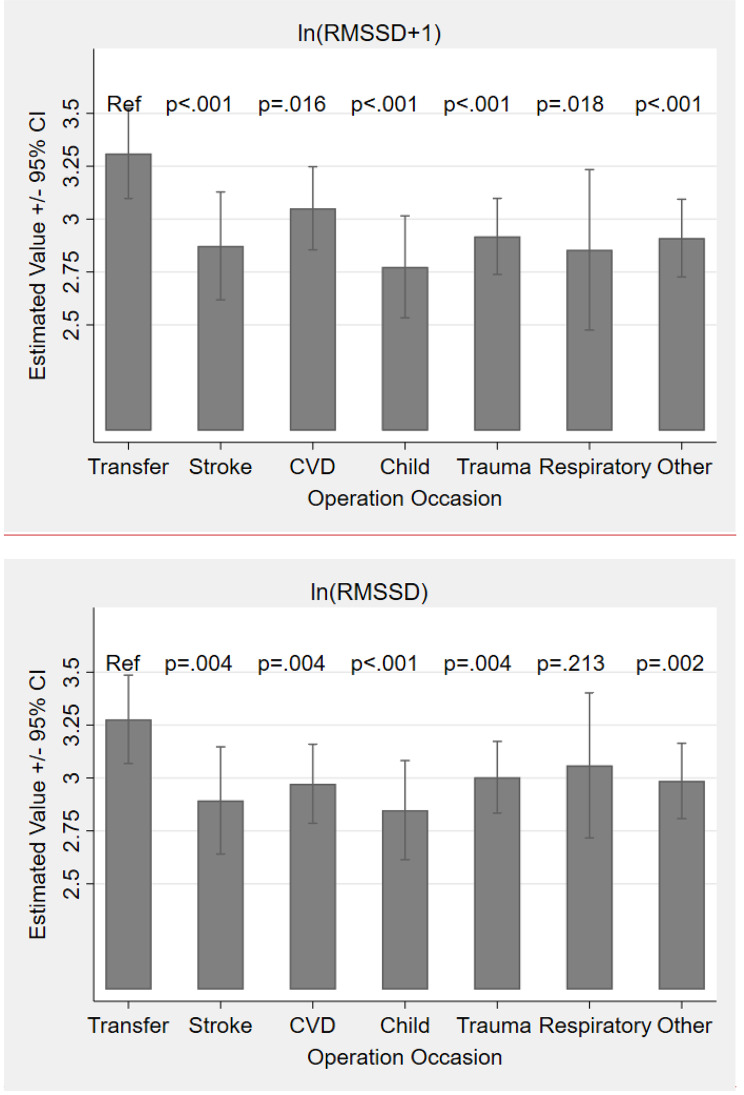
Estimated Effects of Operation Diagnoses on log-transformed RMSSD Note: RMSSD = Root Mean Square of the Successive Differences. Error Bars Represent 95% Confidence Intervals

**Fig. 1b:**
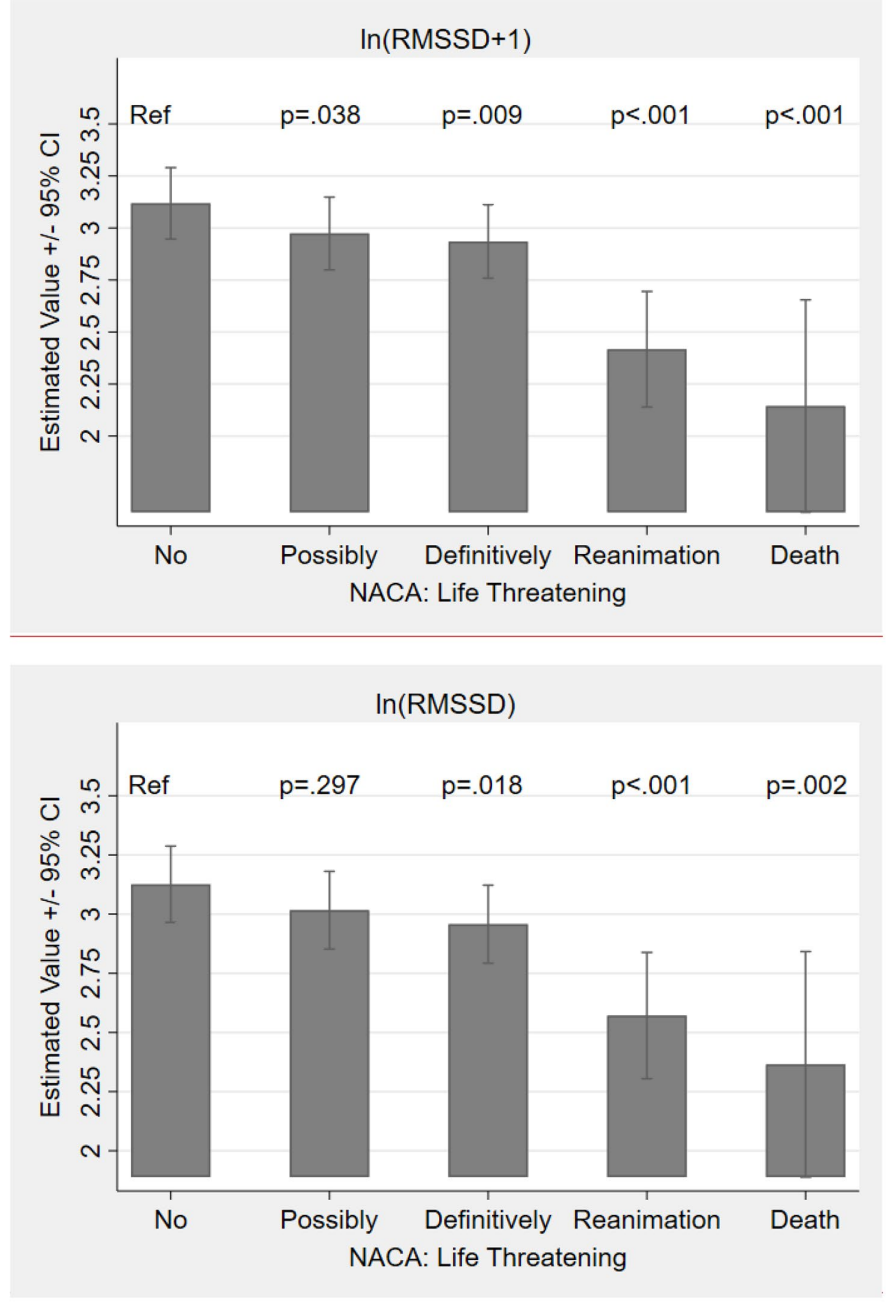
Estimated Effects of the NACA Score on log-transformed RMSSD Note: RMSSD = Root Mean Square of the Successive Differences. Error Bars Represent 95% Confidence Intervals

**Fig. 1c:**
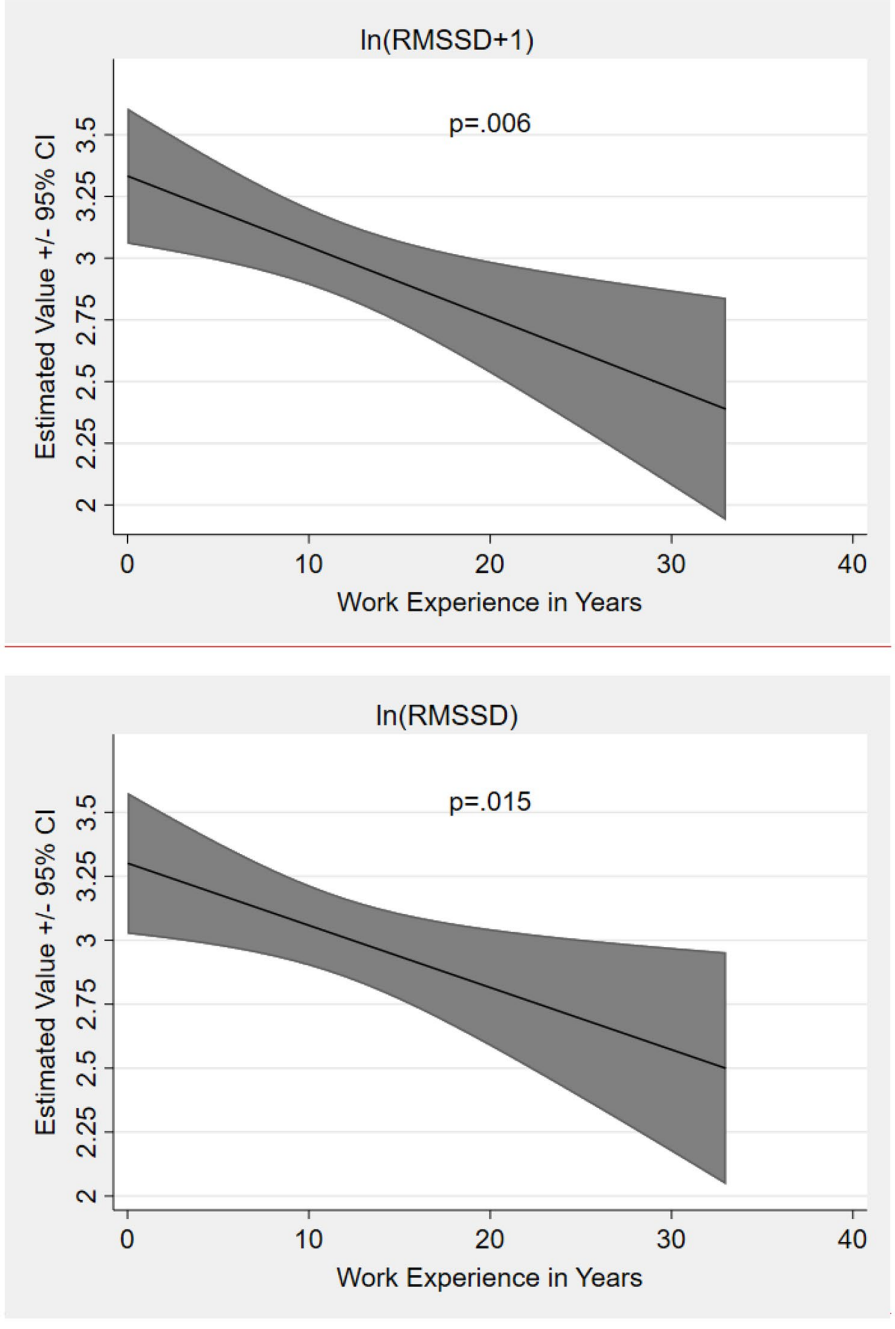
Estimated Effects of the Work Experience in Years on log-transformed RMSSD Note: RMSSD = Root Mean Square of the Successive Differences. Density Represent 95% Confidence Intervals

### Effects on LF/HF

In a similar vein to the model including RMSSD, the random-intercept, fixed-slope models including the repeated measure time variables as well as the fixed effect level 1 variables operation diagnoses, NACA, and work experience showed improved model fit to the null model. The Akaike information criterion of the null model was AIC = 875.27, compared to the model with all predictors AIC_Full Model_= 823.40. Across the different models, the only repeated measure variable with a significant main effect was the operation as indicated by the fixed effect Omnibus test (see Supplementary Material). The fixed effect parameter estimates suggest a higher LF/HF ratio in earlier operations. This indicates higher activity of the sympathetic nervous system.

Interestingly, operation diagnoses showed no significant main effect on the LF/HF ratio in the fixed effect Omnibus test (Table A 1). Some clusters of operation diagnoses reached significance in the fixed effect parameter estimates (Table A 2). Here, the stroke cluster showed the highest sympathetic activation, whereas cardiovascular incidents showed even less sympathetic activation than transfer. However, these effects are deemed to be unsubstantial due to the non-significant Omnibus test.

In line with the RMSSD analysis, the NACA scale showed a significant main effect on the LF/HF ratio as indicated by the fixed effect Omnibus test (Table A 1). The pattern of increased stress by severity was analogous to the effects seen in the RMSSD analysis, yet less pronounced. Reanimation, death, and definitely life-threatening NACA scores reached statistical significance in the fixed effect parameter estimates (Table A 2).

The work experience in years showed significance in the fixed effect Omnibus test. The fixed effect parameter estimates (Table A 2) indicated that the more work experience a physician had accumulated the more sympathetic activation was elicited.

It is noteworthy that the analysis of SDNN did not display any additional information. Results were omitted as they were redundant and less pronounced or revealed no significant main effects.

## Discussion

Over the past years, numerous international studies have shown that rates of emergency service employees’ stress load and stress related illness are exceptionally high [8]. However, there has been little research about the acute stress load and the kind of stressors emergency physicians of the HEMS face and their effects on these physicians’ health. Unexpectedly, the number of operations in one shift did not lead to an increase in objective stress load (autonomic nervous system [1]). For a better comprehension of the objective stress load, patients’ different diagnoses have to be considered. Even though there seem to be no changes in HRV over the course of operations in one shift with an increasing number of operations [1], physiological stress might increase more in diagnoses concerning small children (H1) or with time-critical diagnoses (H2). In addition, the more years of experience in the job, the less physiological stress is activated (H3).

The quality check of the data showed that the first day appeared slightly more stressful (RMSSD) than the second day. This might be explained by EPs wearing ECG devices for the first time and by physicians’ being more accustomed to wearing those devices on the second day. In line with Schöniger et al. [1], the landing phase proved the most stressful time compared to those afterwards, likely due to physicians simultaneously being occupied with medical tasks as well as assisting the pilot during landing. However, in contrast to Schöniger et al. [1], the first operations at the beginning of the day were more stressful (RMSSD, LF/HF) showing a higher activity of the sympathetic nervous system than the following. This decrease might be explained by physiological habituation processes or getting back into the routine.

Concerning the diagnoses clusters and the RMSSD, all operation diagnoses except respiratory incidents were significantly more stressful than a transfer operation. In particular, operation diagnoses concerning children had the strongest significant effect (H1), followed by strokes and cardiovascular diseases as time-critical diagnoses. For the LF/HF, none of the operation diagnoses showed a significant main effect, however, the stroke cluster showed the highest sympathetic activation, whereas respiratory incidents and transfer showed less sympathetic activation. In addition, time-critical life-threatening situations, reanimations and death (NACA) significantly predicted a lower RMSSD. With 14.23% explained variance within, this was the strongest effect out of all of present analyses (H2), suggesting that very severe operation diagnoses impact physicians’ ANS in particular. This pattern of increased stress with likewise increased severity was analogous in the LF/HF to the effects seen in the RMSSD analysis. The present study showed that operation diagnoses concerning children (H1) as well as time-critical diagnoses (H2) are most stressful and have the largest impact on the physicians’ ANS.

Regarding work experience (H3), the present analyses showed a lower RMSSD with increasing work experience. For the LF/HF the analyses indicated that the more work experience a physician had accumulated the more sympathetic activation was elicited. These results are in contrast to what had been expected beforehand but might be explained by three different arguments. Firstly, with increasing work experience emergency physicians might become more and more sensitive to the social consequences of the passing events. Secondly, with increasing work experience additional stressors might be present as a result of physicians holding higher positions in the clinical context that come with greater responsibilities. Hence, the ANS is more stressed and has less recovery phases. Thirdly, with increasing work experience emergency physicians get older and the ANS system less flexible. The present data however cannot answer this question and longitudinal investigations are required to further understanding of this effect.

In contrast to the study conducted by Schöniger et al. [1], in which SDNN showed a significant decrease between the alarm phase and the end of the operation as well as from the first to the third mission, no physiological fatigue indicators and no significant differences for the HRV parameter SDNN could be found. Furthermore, in the present study the first operations proved the most stressful compared to those afterwards, but no indicators for fatigue could be found here either. Compared to standard values, the EPs of this study also showed lower HRV values, which indicates a strong activation of the autonomic nervous system, perhaps caused by a high psychological stress load such as the time pressure induced by the diagnoses over the course of the operations.

### Limitations

The strength of the present study is the measurement of objective stress with markers such as the RMSSD as well as markers for the balance of sympathetic and parasympathetic activation (LF/HF). However, the results are limited to the German rescue station and the Swiss Air-Rescue Rega. In addition, the sample size is quite small. Therefore, a larger sample size as well as investigations with markers for the sympathetic and the parasympathetic activation (cortisol) would present more detailed information on the balance of both stress systems.

## Conclusion

The present study showed that operation diagnoses with children as well as time-critical diagnoses are most stressful and have the largest impact on physicians’ ANS. In addition, a lower RMSSD as well as greater sympathetic activation (LF/HF) with increasing work experience was observed. Across all physicians and independent of work experience the first emergency operations were accompanied by the highest stress levels, which decreased as they habituated over time. Therefore, the assumption that fatigue increases with each additional operation over the course of one shift cannot be attested to in this study.

## Electronic supplementary material

Below is the link to the electronic supplementary material.

Supplementary Material 1

## Data Availability

The datasets generated and/or analyzed during this study can be obtained from the corresponding author on reasonable request.
