# Supplementary material for: Stress of emergency physicians during helicopter operations: impact of patients’ diagnoses, severity of diagnoses, and physicians’ work experience
Source: BMC Emerg Med. 2023 Feb 20;23:20. doi: 10.1186/s12873-023-00786-x (PMC9942287; doi:10.1186/s12873-023-00786-x)
Supplement: Supplementary file 1 — Supplementary Material 1 [file 12873_2023_786_MOESM1_ESM.docx]

**Supplementary Material**

Table A 1

Results of the Fixed Effect Omnibus Tests of Day, Operation, Phase, Deployment Diagnoses, NACA, and Work Experience in Years on LF/HF.

|  | F | Num df | Den df | p |
| --- | --- | --- | --- | --- |
| Day | 3.17 | 1 | 475.71 | .075 |
| Operation | 2.51 | 8 | 468.76 | .011* |
| Phase | 2.48 | 2 | 422.91 | .085 |
| Operation Diagnoses | 1.91 | 6 | 466.90 | .078 |
| NACA | 3.44 | 4 | 475.85 | .009** |
| Work Experience in Years | 6.99 | 1 | 65.76 | .010** |

Note. p ≤ .05*. p ≤ .01**. p ≤ .001***; NACA = National Advisory Committee for Aeronautics Score; LF/HF = Low-to-High Frequency Ratio.

Table A 2

Results of the Fixed Effect Parameter Estimates of Day, Operation, Phase, Operation Diagnoses, NACA, and Work Experience in Years on LF/HF.

| Names | Estimate | SE | df | t | p |
| --- | --- | --- | --- | --- | --- |
| (Intercept) | 6.49 | 0.09 | 274.40 | 69.23 | < .001*** |
| Reference | Day 1 |  |  |  |  |
| Day 2 | -0.14 | 0.08 | 475.71 | -1.78 | .075 |
| Reference | Operation 1 |  |  |  |  |
| Operation 2 | 0.21 | 0.07 | 476.88 | 2.80 | .005** |
| Operation 3 | 0.19 | 0.08 | 476.27 | 2.34 | .020* |
| Operation 4 | 0.09 | 0.09 | 476.58 | 1.06 | .288 |
| Operation 5 | 0.28 | 0.12 | 472.40 | 2.26 | .024 |
| Operation 6 | 0.29 | 0.14 | 469.05 | 2.09 | .038 |
| Operation 7 | 0.45 | 0.15 | 473.81 | 2.95 | .003** |
| Operation 8 | 0.15 | 0.25 | 475.76 | 0.57 | .566 |
| Operation 9 | -0.36 | 0.35 | 474.10 | -1.05 | .295 |
| Reference | Phase 1 |  |  |  |  |
| Phase 2 | 0.11 | 0.06 | 422.91 | 1.88 | .060 |
| Phase 3 | 0.11 | 0.06 | 422.91 | 1.97 | .050* |
| **Operation Diagnoses:** |  |  |  |  |  |
| Reference | Transfer |  |  |  |  |
| Stroke | 0.26 | 0.14 | 474.14 | 1.94 | .053 |
| CVD | -0.04 | 0.11 | 465.33 | -0.34 | .735 |
| Child | 0.20 | 0.13 | 476.29 | 1.55 | .121 |
| Trauma | -0.16 | 0.10 | 476.96 | 0.35 | .106 |
| Respiratory | 0.10 | 0.20 | 471.74 | 0.52 | .606 |
| Other | 0.16 | 0.10 | 480.76 | 1.63 | .104 |
| **NACA  Life Threatening:** |  |  |  |  |  |
| Reference | No |  |  |  |  |
| Possibly | 0.10 | 0.07 | 472.20 | 1.36 | .175 |
| Definitively | 0.20 | 0.07 | 472.64 | 2.79 | .005** |
| Reanimation | 0.33 | 0.13 | 475.86 | 2.50 | .013* |
| Death | 0.61 | 0.26 | 476.96 | 2.34 | .019* |
| **Work Experience in Years** | 0.02 | 0.01 | 65.76 | 2.64 | .010** |

Note. p ≤ .05*. p ≤ .01**. p ≤ .001***; NACA = National Advisory Committee for Aeronautics Score; LF/HF = Low-to-High Frequency Ratio.

Figure A 1a

Estimated Effects of Operation Diagnoses on log-transformed LF/HF


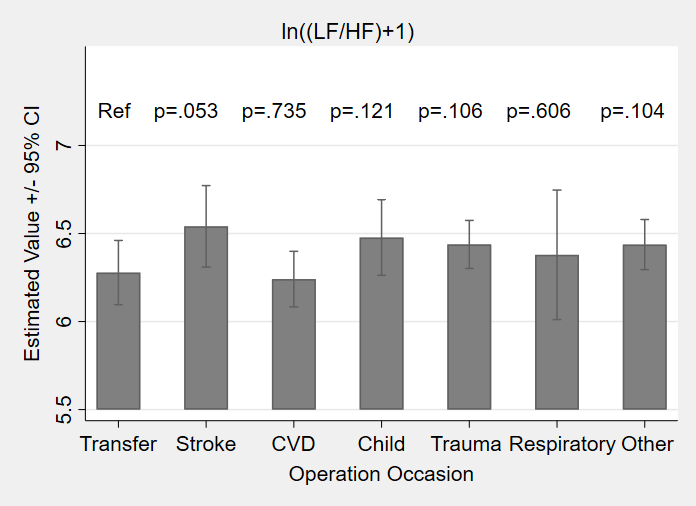


Note. LF/HF = Low-to-High Frequency Ratio. Error Bars Represent 95% Confidence Intervals.

Figure A 1b

Estimated Effects of the NACA Score on log-transformed LF/HF


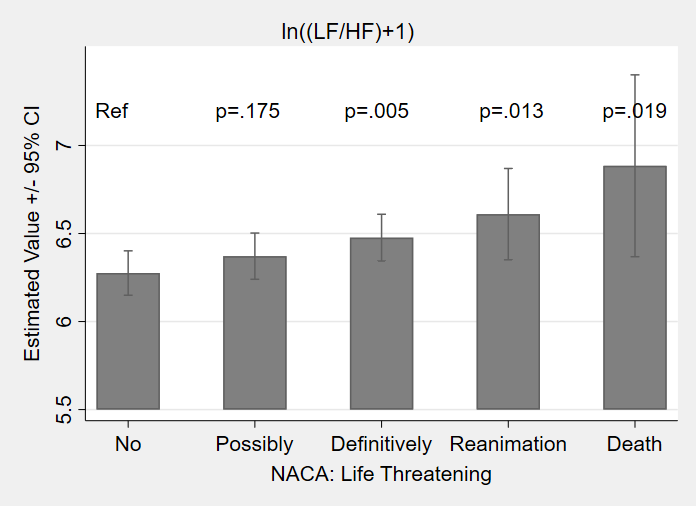


Note. LF/HF = Low-to-High Frequency Ratio. Error Bars Represent 95% Confidence Intervals.

Figure A 1c

Estimated Effects of the Work Experience in Years on log-transformed LF/HF


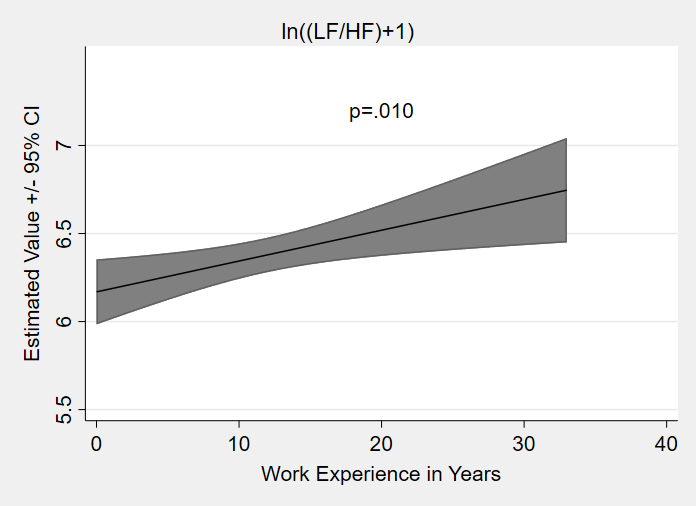


Note. LF/HF = Low-to-High Frequency Ratio. Density Represent 95% Confidence Intervals.
